# Comparative screening identifies gga-miR-204 as a nutrient-responsive miRNA that targets *MAP1LC3B* and promotes infectious bursal disease virus replication in HD11 cells

**DOI:** 10.1016/j.psj.2026.108005

**Published:** 2026-10-21

**Authors:** Shihao Chen, Jiaxing Wang, Qiangzhou Wang, Zhichao Zhang, Hao Bai, Guobin Chang

**Affiliations:** a College of Animal Science and Technology, Yangzhou University, 48 East Wenhui Road, Yangzhou, Jiangsu, 225009, China; b College of Veterinary Medicine, Yangzhou University, Yangzhou, Jiangsu, 225009, China

**Keywords:** Infectious bursal disease virus, gga-miR-204, MAP1LC3B, Nutrient deprivation

## Abstract

Infectious bursal disease virus (IBDV) is a highly contagious immunosuppressive pathogen that causes substantial economic losses to the poultry industry. Although metabolic pathways and key metabolic genes involved in viral replication have been extensively studied, systematic approaches for identifying nutrient-responsive microRNAs (miRNAs) that functionally regulate IBDV replication remain limited. In this study, amino acid- and glucose-starvation models were established in chicken HD11 macrophages and integrated with IBDV infection-associated miRNA profiling to comparatively screen for nutrient-responsive miRNAs potentially involved in viral replication. Short-term nutrient deprivation altered LC3-associated molecular phenotypes and reduced IBDV VP2 protein expression. Small RNA sequencing revealed that nutrient starvation and IBDV infection induced distinct but partially overlapping miRNA expression profiles, with predicted target genes enriched mainly in metabolic and stress-response pathways. gga-miR-204 was upregulated under amino acid and glucose starvation but downregulated following IBDV infection. Functional assays showed that gga-miR-204 overexpression increased viral RNA levels, VP2 protein expression, and infectious virus production, whereas inhibition of endogenous gga-miR-204 suppressed IBDV replication. Reporter assays supported targeting of the chicken *MAP1LC3B* 3′ untranslated region by gga-miR-204, and mimic treatment reduced LC3-II abundance in HD11 cells. *MAP1LC3B* silencing increased VP2 abundance, whereas *MAP1LC3B* overexpression decreased it. These findings identify gga-miR-204 as a nutrient-responsive miRNA that promotes IBDV replication in HD11 cells and support *MAP1LC3B* as a candidate target with functional effects on VP2 abundance. Whether *MAP1LC3B* mediates the proviral effect of gga-miR-204 remains to be established. This comparative screening approach provides a starting point for identifying nutrient-responsive miRNAs and investigating their potential roles in linking cellular metabolic state to IBDV replication.

## Introduction

Infectious bursal disease virus (IBDV) is the causative agent of infectious bursal disease and primarily infects B lymphocytes in the bursa of Fabricius, while also targeting immune cells such as macrophages. IBDV infection causes severe immunosuppression and increases the risk of secondary infections (Guo et al., 2026; Niu et al., 2024; Zhang et al., 2023). Although vaccination has been widely used for the prevention and control of IBD, the continuous emergence of variant and very virulent IBDV (vvIBDV) strains remains a major threat to the poultry industry (Dulwich et al., 2020; Zhang et al., 2022). Elucidating how host-cell states and regulatory molecules influence IBDV replication is therefore important for understanding viral pathogenesis and developing improved host-directed control strategies.

Viral replication is highly dependent on host-cell energy supply, nutrient substrates, and biosynthetic machinery (Girdhar et al., 2021). IBDV infection can reprogram host metabolic networks and affect autophagy-related pathways (Song et al., 2025; Wang et al., 2025b, 2017). Nutrient deprivation, as a defined form of cellular stress, can influence viral replication by restricting metabolic resources and reshaping stress-responsive signaling networks (Jassey et al., 2023; Li et al., 2019). However, nutrient stress does not necessarily produce a uniformly antiviral or proviral response. Its overall effect reflects the balance among metabolic restriction, cellular adaptation, autophagy-associated processes, and other host pathways that may exert opposing effects on viral replication (He et al., 2018; Onishi et al., 2019). How these functionally divergent responses collectively influence IBDV replication remains poorly understood.

MicroRNAs (miRNAs) are important post-transcriptional regulators that primarily modulate gene expression through sequence-specific interactions with the 3′ untranslated regions (3′ UTRs) of target mRNAs. They participate extensively in metabolic adaptation, stress responses, autophagy, and viral infection (Wang et al., 2025c). Several noncoding RNAs have been implicated in host immune regulation during IBDV infection (Fu et al., 2018a, 2018b). Stress-induced immunosuppression can also alter miRNA expression and affect immune responses to IBDV vaccination through regulatory networks involving TGF-β, MAPK, and miR-27b-3p/SOCS3 (Ma et al., 2023; Wu et al., 2021). In addition, nutrient-responsive miRNAs can regulate genes associated with autophagy, apoptosis, and metabolism (Li et al., 2016; Mukherjee et al., 2016). Although metabolic pathways and key metabolic genes involved in viral replication have been extensively investigated, systematic approaches for identifying nutrient-responsive miRNAs that functionally regulate IBDV replication remain limited. Moreover, it is unclear whether the effects of individual nutrient-responsive miRNAs on viral replication align with the overall effect of nutrient deprivation.

In this study, amino acid- and glucose-starvation models were established in chicken HD11 macrophages and integrated with IBDV infection-associated miRNA profiling to comparatively screen for nutrient-responsive miRNAs potentially involved in viral replication. Using this approach, gga-miR-204-5p (hereafter referred to as gga-miR-204) was identified as a miRNA that was upregulated by nutrient deprivation but downregulated following IBDV infection. We therefore investigated its effects on IBDV replication and its potential target genes. Functional analyses showed that gga-miR-204 promoted IBDV replication and reduced LC3-II abundance, while reporter assays supported its targeting of the 3′ UTR of chicken MAP1LC3B, which encodes microtubule-associated protein 1 light chain 3 beta, an ATG8-family autophagy-related protein. *MAP1LC3B* silencing increased VP2 abundance, whereas its overexpression decreased it. These findings support a candidate gga-miR-204–*MAP1LC3B* regulatory relationship, although whether *MAP1LC3B* mediates the proviral effect of gga-miR-204 remains unresolved. This comparative screening approach provides a starting point for identifying additional nutrient-responsive miRNAs and investigating their potential roles in linking cellular metabolic state to IBDV replication.

## Materials and methods

### Cells and virus

The chicken macrophage cell line HD11 was routinely maintained in our laboratory. Cells were cultured in Dulbecco’s modified Eagle’s medium (DMEM; Gibco, USA) containing 10% fetal bovine serum (FBS; Gibco), 100 U/mL penicillin, and 100 μg/mL streptomycin. Cultures were kept at 37°C under a humidified atmosphere with 5% CO₂. The IBDV LM strain, kindly provided by Professor Jue Liu from Yangzhou University, was amplified in our laboratory before use. For infection experiments, HD11 cells were seeded one day before infection and inoculated with the IBDV LM strain at a multiplicity of infection (MOI) of 1. Following adsorption for 1 h at 37 °C, the inoculum was removed, the cells were washed once with phosphate-buffered saline (PBS), and fresh DMEM supplemented with 1% FBS was added as the maintenance medium. Samples for the functional assays were collected at 24 h post-infection.

### Amino acid and glucose starvation

For starvation assays, HD11 cells were transferred to nutrient-deficient media according to the experimental design. Amino acid starvation was performed by washing the cells with PBS followed by culture in amino acid-free Krebs-Ringer buffer (PB180347, Procell, Wuhan, China), whereas glucose deprivation was conducted using glucose-free DMEM (PM150270, Procell, Wuhan, China). The treatments were maintained for the indicated time periods, with HD11 cells cultured in complete DMEM serving as controls.

### Small RNA sequencing (sRNA-seq) and bioinformatics analysis

For sRNA-seq, total RNA was extracted using TRIzol reagent (Invitrogen, USA) from three independent biological replicates per condition: untreated control, amino acid starvation for 4 h, glucose starvation for 4 h, and IBDV infection at an MOI of 1 for 24 h. The nutrient starvation and IBDV infection treatments were performed as described in our previous study (Wang et al., 2025b). Only RNA samples meeting the required quality criteria were used for small RNA library construction. Library preparation and Illumina sequencing were performed by Gene Denovo Biotechnology Co., Ltd. (Guangzhou, China). The generated reads were first cleaned by removing adaptor contamination and low-quality sequences. Differentially expressed miRNAs were identified using thresholds of |log₂ fold change| ≥ 1 and adjusted *P* < 0.05. P values were adjusted using the Benjamini–Hochberg procedure to control the false discovery rate. Principal component analysis (PCA), clustering heatmaps, and Venn diagrams were generated using R version 4.5.2. Potential mRNA targets of the differentially expressed miRNAs were predicted using TargetScan (https://www.targetscan.org/vert_80/), and the predicted target genes were subjected to Kyoto Encyclopedia of Genes and Genomes (KEGG) pathway enrichment analysis.

### RT-qPCR

Reverse transcription was performed using different systems for mRNA and miRNA assays. For mRNA detection, cDNA was generated with HiScript SuperMix for qPCR (+gDNA wiper) (R323-01, Vazyme, China), whereas miRNA reverse transcription was performed using the Mir-X miRNA First-Strand Synthesis Kit (Takara, Japan). Quantitative PCR was carried out on a CFX Connect Real-Time PCR Detection System (Bio-Rad, USA) with ChamQ SYBR qPCR Master Mix (Q341-02, Vazyme, China). Each reaction was prepared in a final volume of 20 µL, consisting of 10 µL of 2 × ChamQ SYBR qPCR Master Mix, 0.4 µL each of forward and reverse primers (10 µM), 1 µL of cDNA template, and nuclease-free water. The amplification program included an initial denaturation at95 °C for 3 min, followed by 40 cycles of 95 °C for 15 s and 60 °C for 15 s. β-actin and U6 were used as internal controls for mRNA and miRNA quantification, respectively. The universal reverse primer and U6 primer used for miRNA detection were supplied with the Mir-X kit. The primer sequences were as follows: VP2 forward, 5′-ACCGGCACCGACAACCTT-3′ and reverse, 5′-CCCTGCCTGACCACCACTT-3′; *MAP1LC3B* forward, 5′- AGGTACAAGGGGGAGAAGCA-3′ and reverse, 5′- GTCCGTTCACCAGGAGGAAG-3′; β-actin forward, 5′-GAGAAATTGTGCGTGACATCA-3′ and reverse, 5′-CCTGAACCTCTCATTGCCA-3′; gga-miR-204 forward, 5′-TTCCCTTTGTCATCCTATGCCT-3′; gga-miR-1574-5p forward, 5′-TCGCTGTGACTTCTCCTTGT-3′.

### Cell Transfection

The gga-miR-204 mimic and inhibitor, negative control (NC), and chicken *MAP1LC3B*-targeting siRNA were purchased from RiboBio Co., Ltd. (Guangzhou, China). HD11 cells were transfected using the Mirus TransIT-X2® Dynamic Delivery System (Mirus Bio, USA) as recommended by the manufacturer. For LC3B overexpression experiments, cells were transfected with the pcDNA3.1-GFP-LC3B, with the corresponding empty vector used as a control.

### Western blot analysis

Western blotting was performed as previously described, with minor modifications(Wang et al., 2025a). Briefly, cells were harvested and lysed in RIPA buffer supplemented with protease inhibitors, and total protein concentrations were determined by BCA assay. Equal quantities of protein were separated by SDS-PAGE and transferred to nitrocellulose membranes. After blocking with 5% non-fat milk, the membranes were incubated at 4°C overnight with primary antibodies against VP2, LC3B (CST, #2741), p62 (CST, #5114), GFP (ABclonal, #AE078), β-actin (ABclonal, #AC026), or Tubulin (MBL, #PM054). The VP2 monoclonal antibody, provided by Professor Shijun Zheng from China Agricultural University, was diluted 1:1,000. HRP-conjugated goat anti-mouse and goat anti-rabbit IgG antibodies (NCM, Suzhou, China; 1:10,000) were used as secondary antibodies. Signals were developed using ECL reagent and quantified in ImageJ.

### Viral titration by TCID₅₀ assay

Viral titers were measured as previously described with slight modifications(Chen et al., 2025a). HD11 cells were transfected with gga-miR-204 mimic, mimic negative control, gga-miR-204 inhibitor, or inhibitor negative control, followed by IBDV infection 24 h later. Culture supernatants were collected at 24 h post-infection, clarified by low-speed centrifugation, and preserved at low temperature before analysis. For titration, serial dilutions of each supernatant were added to DF-1 cells seeded in 96-well plates, with multiple replicate wells for each dilution. After 1 h of adsorption, the inoculum was removed, cells were washed once with PBS, and maintenance medium was added. Following incubation at 37 °C for 72 h, cytopathic effects were examined microscopically, and viral titers were calculated according to the Reed–Muench TCID₅₀ method.

### Immunofluorescence assay

HD11 cells cultured onto coverslips were processed after the indicated treatments. Cells were fixed with 4% paraformaldehyde, permeabilized with 0.1% Triton X-100, and blocked with 5% BSA before incubation with the anti-VP2 primary antibody. After labeling with a fluorophore-conjugated secondary antibody, nuclei were counterstained with DAPI. Images were obtained using an Olympus IX71 fluorescence microscope, and fluorescence signals were measured in ImageJ.

### Plasmid construction

The pcDNA3.1(+)-chLC3B-GFP, pcDNA3.1(+)-GFP, and the pmir-GLO dual-luciferase reporter vector were available in our laboratory. To generate *MAP1LC3B* 3′UTR reporter constructs, the wild-type (WT) and mutant (Mut) fragments were amplified and cloned into the pmir-GLO vector. The reporter backbone was prepared by double digestion with Nhe I and Not I-HF. For the WT construct, total RNA extracted from HD11 cells was reverse-transcribed into cDNA, which served as the template for PCR amplification of the *MAP1LC3B* 3′UTR. The Mut 3′UTR fragment was synthesized by Dynegene Technologies and amplified using the same primer set. The amplified WT and Mut PCR products were then assembled with the linearized pmir-GLO vector by homologous recombination. The primers used for amplification were as follows: forward, 5′-TAGTTGTTTAAACGAGCTCGCTAGCGGATGTCACCTATGCTCTCAGTAC-3′; reverse, 5′-CCTGCAGGTCGACTCTAGAGATCTCAGCGAGCTGCAGA-3′.

### Dual-luciferase reporter assay

293T cells were seeded into 12-well plates and used for transfection at approximately 80% confluence. The pmir-LC3B-3′UTR WT or Mut plasmids were co-transfected with NC mimics or gga-miR-204 mimics, using 500 ng plasmid and 50 nM mimic per well Luciferase activities were examined 36 h later following the manufacturer’s instructions. Firefly luciferase activity was normalized to Renilla luciferase activity.

### Statistical analysis

All experiments were independently repeated at least three times. Data are shown as the mean ± SD and were analyzed in GraphPad Prism. Student’s t-test was used for two-group comparisons, and one-way ANOVA was applied for multiple-group analyses. P < 0.05 was considered statistically significant.

## Results

### Establishment of nutrient starvation models and their effects on IBDV replication

To investigate the effects of nutrient starvation on IBDV infection, GS and AAS conditions were established in the chicken macrophage cell line HD11 as previously described (Wang et al., 2025b). Cells were cultured in serum-free glucose-free DMEM (GS) or Krebs–Ringer buffer (AAS) for different durations, and the expression of the autophagy-related proteins LC3 and p62 was examined by Western blotting. The results showed that AAS treatment significantly increased the LC3-II/LC3-I ratio at 6 h (*P* < 0.05), accompanied by an apparent decrease in p62 protein levels, suggesting an autophagy-related response under amino acid starvation (Fig. 1A). In contrast, GS treatment significantly increased the LC3-II/LC3-I ratio at 2 and 4 h (*P* < 0.05), followed by a decline at 6 h. p62 showed apparent accumulation at 4 h, suggesting a different pattern of changes in autophagy-related markers under glucose starvation (Fig. 1B). To assess the effect of nutrient starvation on IBDV replication, HD11 cells were infected with IBDV under nutrient-deficient conditions. Under GS conditions, LC3-II levels were increased, whereas VP2 protein expression was reduced compared with the IBDV-infected control group (Fig. 1C). Similarly, AAS using Krebs buffer significantly decreased VP2 protein expression compared with IBDV-infected cells cultured in DMEM, accompanied by changes in LC3-II levels (Fig. 1D). These results indicate that short-term nutrient starvation induces LC3-associated responses in HD11 cells and suppresses IBDV protein expression.

**Fig. 1:**
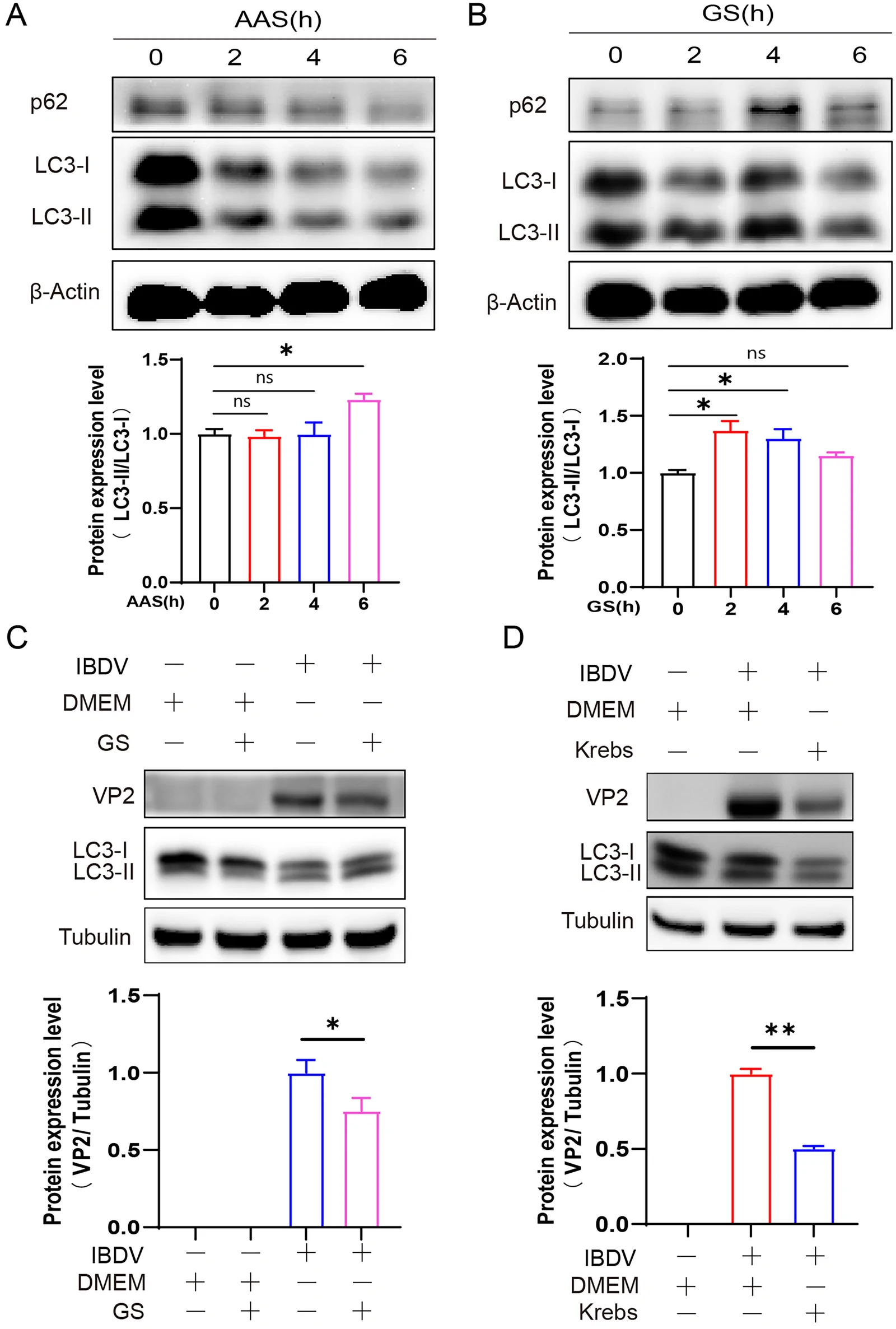
Nutrient starvation alters LC3-associated responses and reduces IBDV VP2 expression in HD11 cells. (A, B) HD11 cells were subjected to amino acid starvation (AAS; A) or glucose starvation (GS; B) for 0, 2, 4, or 6 h. LC3 and p62 were analyzed by Western blotting, and LC3-II/LC3-I ratios were quantified. (C) HD11 cells were infected with IBDV under normal or glucose-free conditions. (D) IBDV-infected cells were cultured in DMEM or Krebs buffer. In panels C and D, VP2 and LC3 were analyzed by Western blotting, and VP2 levels were quantified relative to tubulin. Data are presented as mean ± SD from three independent experiments. *P < 0.05; **P < 0.01; ns, not significant.

### miRNA expression profiling under nutrient stress and IBDV infection

To characterize the effects of nutrient stress and IBDV infection on host miRNA expression, HD11 cells were assigned to control (CTRL), AAS, GS, or IBDV infection groups, followed by miRNA sequencing and bioinformatic analysis (Fig. 2A). Principal component analysis (PCA) showed distinct clustering among treatment groups, with PC1 and PC2 explaining 31% and 9.9% of the total variance, respectively (Fig. 2B). The IBDV group was clearly separated from the CTRL group, whereas the GS group clustered closer to CTRL, suggesting greater differences from CTRL following IBDV infection than following short-term glucose starvation. Differential expression analysis identified 35 upregulated and 23 downregulated miRNAs in the AAS group, 40 upregulated and 18 downregulated miRNAs in the GS group, and 71 upregulated and 143 downregulated miRNAs in the IBDV group compared with CTRL (Fig. 2C). Thus, IBDV infection was associated with a larger number of differentially expressed miRNAs, whereas nutrient starvation caused relatively moderate but treatment-specific changes. KEGG enrichment analysis of predicted target genes showed that targets of AAS-responsive miRNAs were mainly enriched in metabolic pathways, lysosome, SNARE-mediated vesicular transport, and viral protein interaction with cytokine and cytokine receptor pathways (Fig. 2D). Targets of GS-responsive miRNAs were enriched in metabolic pathways, spliceosome, peroxisome, and arginine and proline metabolism (Fig. 2E), whereas targets of IBDV-responsive miRNAs were enriched in metabolic pathways, protein processing in the endoplasmic reticulum, spliceosome, and fluid shear stress and atherosclerosis (Fig. 2F). Collectively, these results indicate that nutrient starvation and IBDV infection reshape the miRNA expression landscape in HD11 cells, with metabolic pathways represented among the enriched pathways of predicted miRNA targets in all three comparisons.

**Fig. 2:**
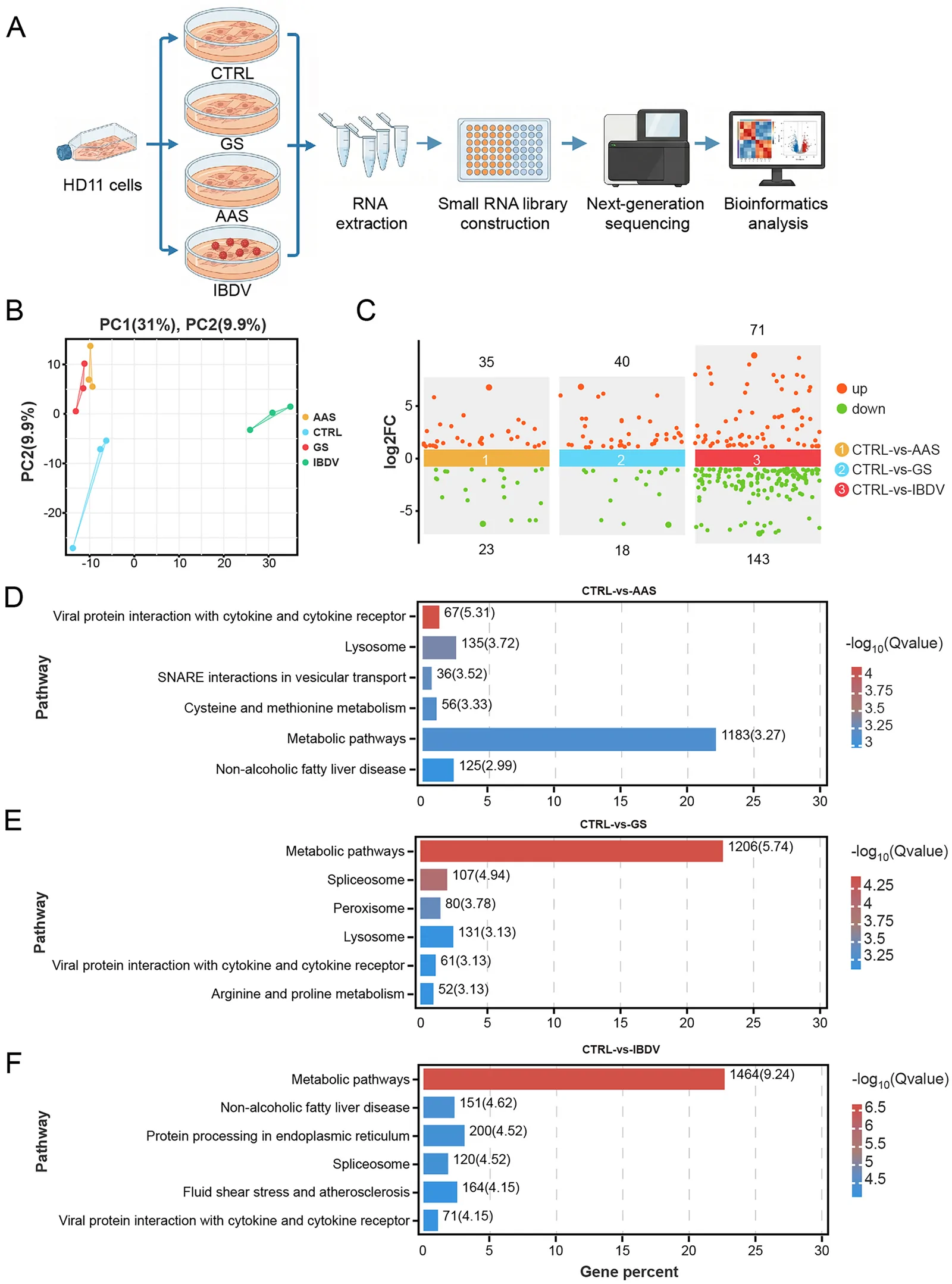
miRNA expression profiles of nutrient-starved and IBDV-infected HD11 cells. (A) Experimental workflow for small RNA sequencing of control (CTRL), AAS, GS, and IBDV-infected cells. (B) Principal component analysis of miRNA expression profiles. (C) Differentially expressed miRNAs in the AAS, GS, and IBDV groups compared with CTRL. Red and green indicate upregulated and downregulated RNAs, respectively. (D–F) KEGG enrichment analysis of predicted target genes of differentially expressed miRNAs in the AAS (D), GS (E), and IBDV (F) comparisons.

### gga-miR-204 is a candidate miRNA responsive to nutrient stress and IBDV infection

To identify candidate miRNAs involved in nutrient stress and IBDV infection, differentially expressed miRNAs were subjected to overlap analysis. Although many treatment-specific miRNAs were identified in the CTRL-vs-AAS, CTRL-vs-GS, and CTRL-vs-IBDV comparisons, a subset was shared among groups (Fig. 3A). AAS and GS shared 5 differentially expressed miRNAs, whereas IBDV shared 12 and 10 miRNAs with AAS and GS, respectively, indicating partial overlap between nutrient starvation- and infection-associated miRNA responses. A heatmap showed distinct miRNA expression patterns among treatment groups (Fig. 3B). Notably, gga-miR-204, gga-miR-1574-5p, gga-miR-211, and several novel miRNAs were differentially expressed across treatments, indicating their potential involvement in nutrient stress and IBDV infection. RT-qPCR validation showed that gga-miR-204 was significantly upregulated after 4 h of AAS or GS treatment (*P* < 0.01), but significantly downregulated at 24 h post-IBDV infection (*P* < 0.01) (Fig. 3C). Similarly, gga-miR-1574-5p was upregulated under AAS and GS conditions but downregulated at the examined time points during IBDV infection (Fig. 3D). These results were consistent with the sequencing data and identified gga-miR-204 and gga-miR-1574-5p as candidates exhibiting opposite expression responses to nutrient starvation and IBDV infection. Given its relatively high abundance among the miRNAs detected by sequencing and the conservation of its seed sequence across chickens and mammals, we focused on gga-miR-204 in the present study to investigate its role in IBDV replication in HD11 cells.

**Fig. 3:**
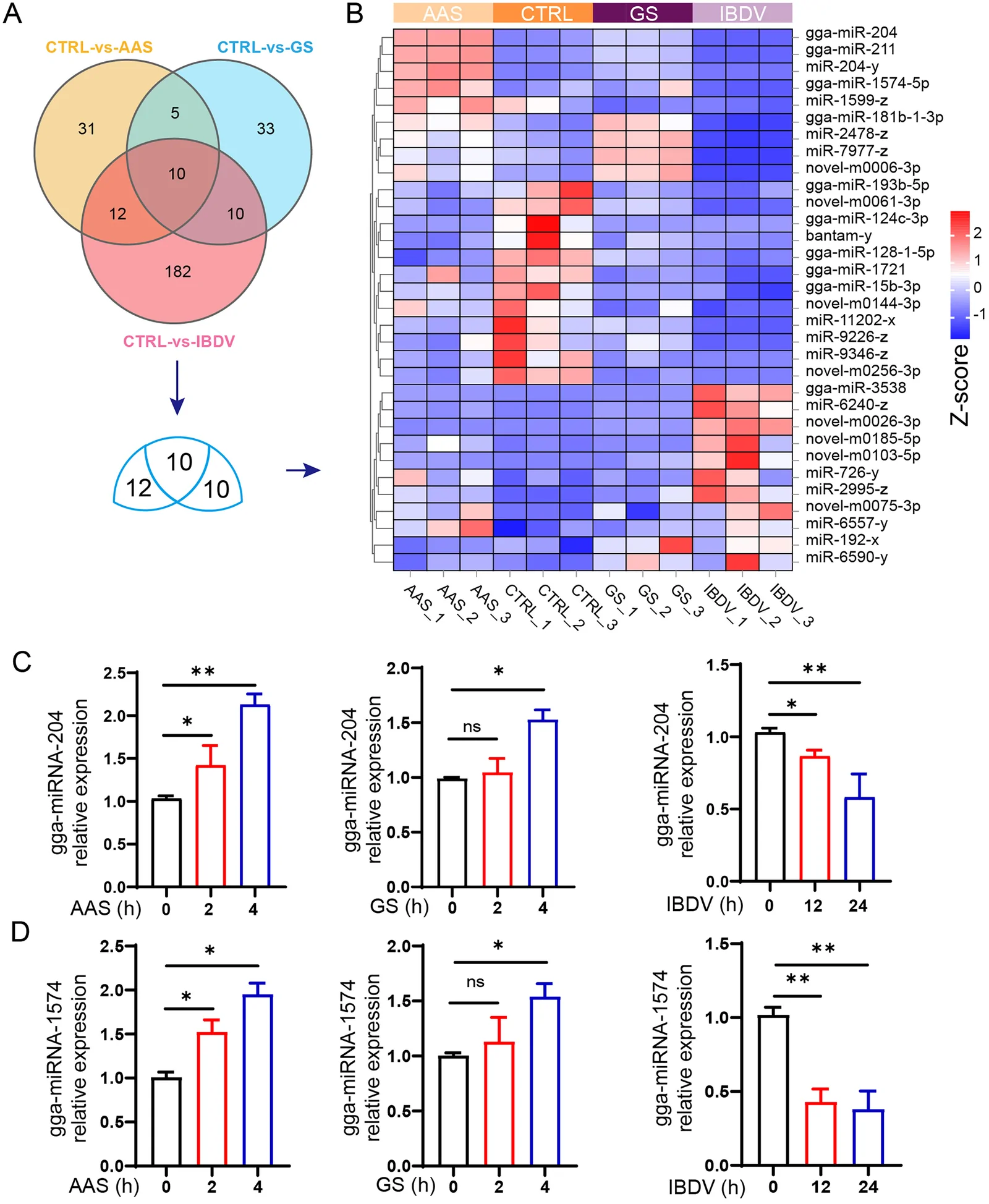
Identification and validation of nutrient- and IBDV-responsive miRNAs. (A) Venn analysis of differentially expressed miRNAs among the AAS, GS, and IBDV comparisons relative to CTRL. The pairwise-only overlaps comprised 5 miRNAs for AAS–GS, 12 for AAS–IBDV, and 10 for GS–IBDV, excluding the three-way intersection. An additional 10 miRNAs were shared by all three comparisons. (B) Heatmap of the 32 miRNAs selected in panel A (n = 3 biological replicates/group). Expression values were scaled to row-wise Z-scores. miRNAs were hierarchically clustered using Euclidean distance and complete linkage. (C, D) RT-qPCR validation of gga-miR-204 (C) and gga-miR-1574-5p (D) following AAS, GS, or IBDV infection at the indicated time points. Data are presented as mean ± SD from three independent experiments. *P < 0.05; **P < 0.01; ns, not significant.

### gga-miR-204 promotes IBDV replication in HD11 cells

To determine the role of gga-miR-204 during IBDV infection, HD11 cells were transfected with a gga-miR-204 mimic and then infected with IBDV. gga-miR-204 overexpression had little effect on VP2 RNA expression at 12 h post-infection but significantly increased VP2 RNA levels at 24 h post-infection (Fig. 4A). Dose-dependent analysis showed that VP2 RNA expression increased with increasing concentrations of gga-miR-204 mimic, with significant upregulation observed in the 30 and 50 nmol/L groups (Fig. 4B). Consistently, Western blotting showed a concentration-dependent increase in VP2 protein expression, which was significant in the 50 nmol/L group (Fig. 4C). To further determine whether gga-miR-204 affects productive IBDV replication, viral titers were measured after modulation of gga-miR-204 expression. Overexpression of gga-miR-204 significantly increased the relative IBDV titer compared with the NC mimic group, whereas inhibition of endogenous gga-miR-204 significantly decreased the viral titer compared with the NC inhibitor group (Fig. 4D). Immunofluorescence assays further confirmed these results, showing enhanced VP2 fluorescence signals after gga-miR-204 overexpression, particularly at higher mimic concentrations (Fig. 4E). Quantification showed significantly increased fluorescence intensity in the 30 and 50 nmol/L groups (*P* < 0.05). Together, these results indicate that gga-miR-204 promotes IBDV replication in HD11 cells in a time- and dose-dependent manner.

**Fig. 4:**
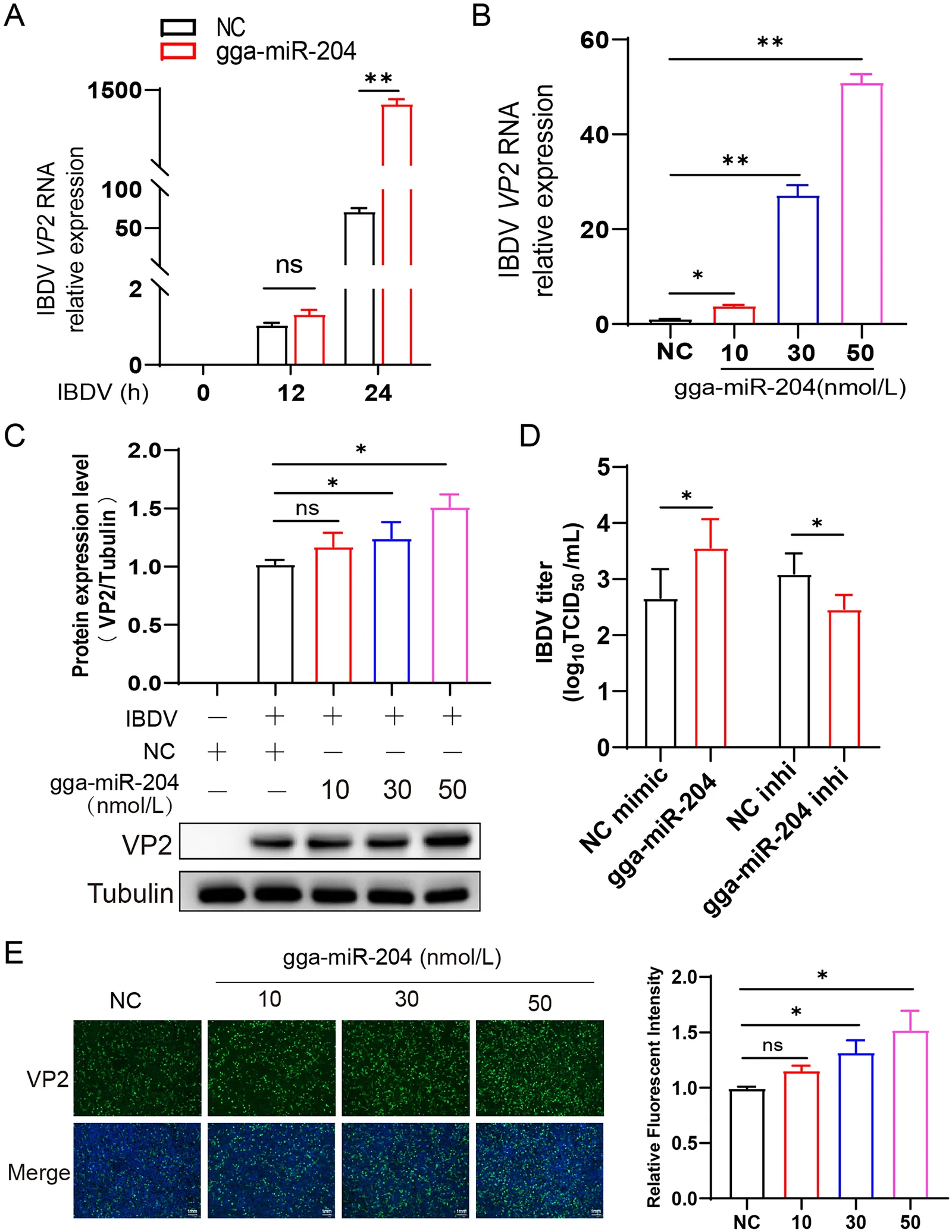
gga-miR-204 promotes IBDV replication in HD11 cells. (A) VP2 RNA levels after transfection with gga-miR-204 mimic or negative control (NC) mimic, followed by IBDV infection. (B, C) Dose-dependent effects of gga-miR-204 mimic on VP2 RNA levles (B) and protein (C) expression. (D) IBDV titers following transfection with gga-miR-204 mimic, inhibitor, or the corresponding controls and subsequent IBDV infection. (E) Immunofluorescence analysis and quantification of VP2 after gga-miR-204 mimic transfection. VP2 is shown in green and nuclei in blue. Data are presented as mean ± SD from three independent experiments. *P < 0.05; **P < 0.01; ns, not significant.

### gga-miR-204 directly targets MAP1LC3B and reduces LC3B protein levels

To investigate the mechanism underlying gga-miR-204-mediated promotion of IBDV replication, its putative mRNA targets were predicted using TargetScan (https://www.targetscan.org/vert_80/). *MAP1LC3B*, was identified as a potential target of gga-miR-204 (Fig. 5A). KEGG enrichment analysis showed that the predicted targets were mainly associated with lysosome, apoptosis, MAPK signaling, SNARE-mediated vesicular transport, and metabolism-related pathways (Fig. 5B), suggesting a possible role of gga-miR-204 in autophagy and cellular stress regulation. Sequence alignment revealed a conserved binding site for gga-miR-204 in the chicken *MAP1LC3B* 3′ UTR, which was also conserved among the examined *MAP1LC3B* orthologs from different species (Fig. 5C). Dual-luciferase reporter assays showed that gga-miR-204 significantly reduced the activity of the wild-type chicken *MAP1LC3B* 3′ UTR reporter but not that of the mutant reporter (Fig. 5D), supporting sequence-specific targeting of this site. Dose-dependent assays further showed that gga-miR-204 mimic gradually reduced wild-type reporter activity, with significant inhibition at 30 and 50 nmol/L (*P* < 0.01) (Fig. 5E), whereas the mutant reporter was unaffected (Fig. 5F). Although gga-miR-204 increased endogenous *MAP1LC3B* mRNA levels (Fig. 5G), it reduced LC3B abundance normalized to β-actin in a concentration-dependent manner (Fig. 5H). Together, these results support direct targeting of the chicken *MAP1LC3B* 3′ UTR by gga-miR-204 and show reduced LC3B abundance following mimic treatment.

**Fig. 5:**
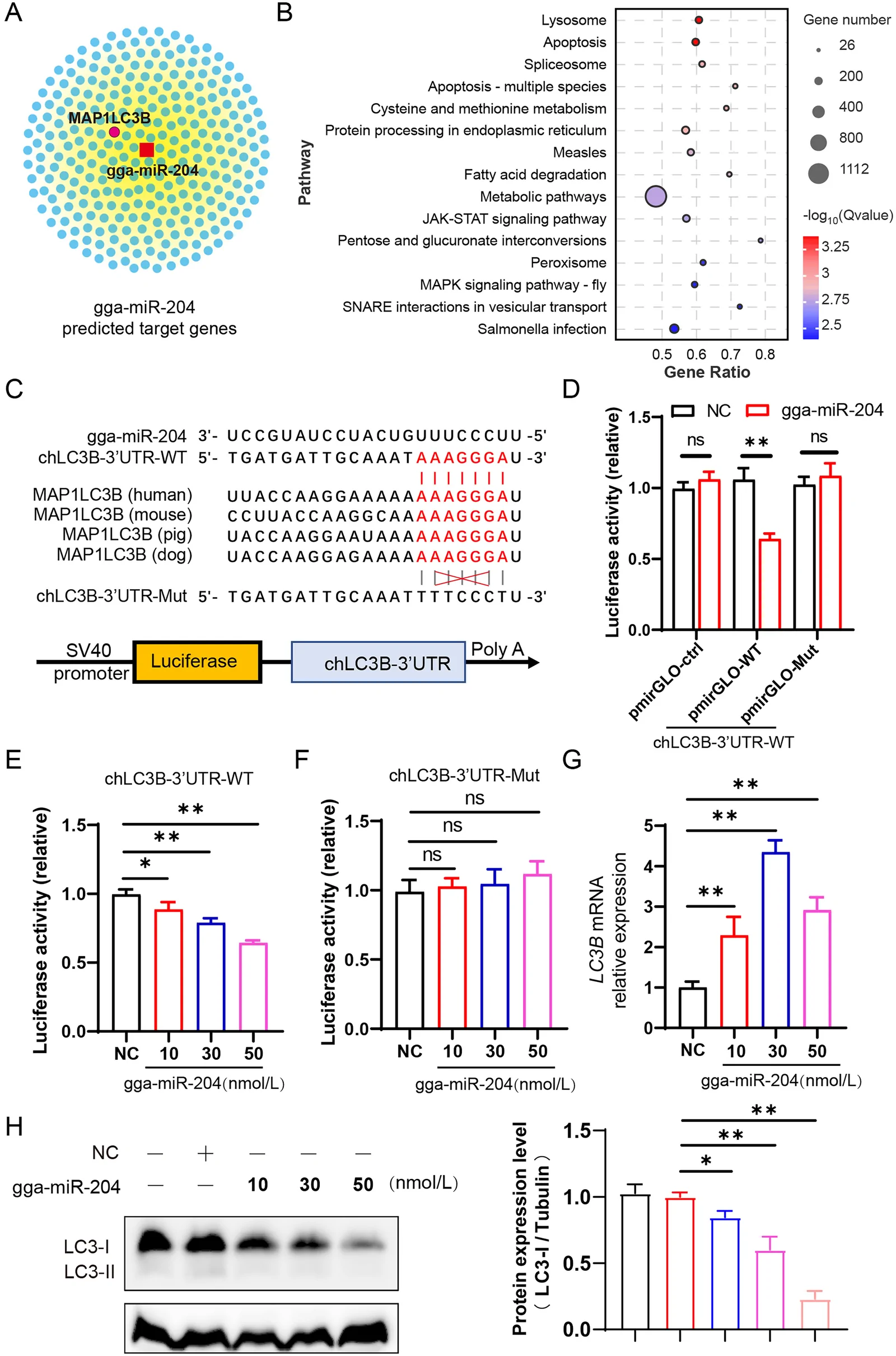
gga-miR-204 directly targets the chicken MAP1LC3B 3′ UTR. (A) Bioinformatic prediction of MAP1LC3B as a potential gga-miR-204 target using TargetScan (https://www.targetscan.org/vert_80/). (B) KEGG pathway enrichment analysis of predicted gga-miR-204 target genes. (C) Sequence alignment showing the predicted gga-miR-204-binding site in the chicken MAP1LC3B 3′ UTR and its conservation among the examined species, together with the corresponding wild-type (WT) and mutant (Mut) reporter sequences. (D) Luciferase activities of the control, MAP1LC3B-3′ UTR-WT, and MAP1LC3B-3′ UTR-Mut reporters following transfection with the gga-miR-204 mimic. (E, F) Dose-dependent effects of the gga-miR-204 mimic on the activities of the WT (E) and Mut (F) reporters. (G, H) Effects of increasing concentrations of the gga-miR-204 mimic on MAP1LC3B mRNA levels (G) and LC3 protein abundance normalized to β-actin (H). Data are presented as mean ± SD from three independent experiments. *P < 0.05; **P < 0.01; ns, not significant.

### gga-miR-204-mediated promotion of IBDV replication is associated with LC3B regulation

To clarify the relationship between gga-miR-204 and LC3B during IBDV infection, gain- and loss-of-function assays were performed. Overexpression of gga-miR-204 increased VP2 protein expression in a concentration-dependent manner, particularly at 30 and 50 nmol/L, accompanied by changes in LC3-II levels (Fig. 6A). Conversely, inhibition of endogenous gga-miR-204 reduced VP2 expression while increasing LC3-II levels (Fig. 6B), suggesting that gga-miR-204 facilitates IBDV replication and is associated with LC3 regulation.

**Fig. 6:**
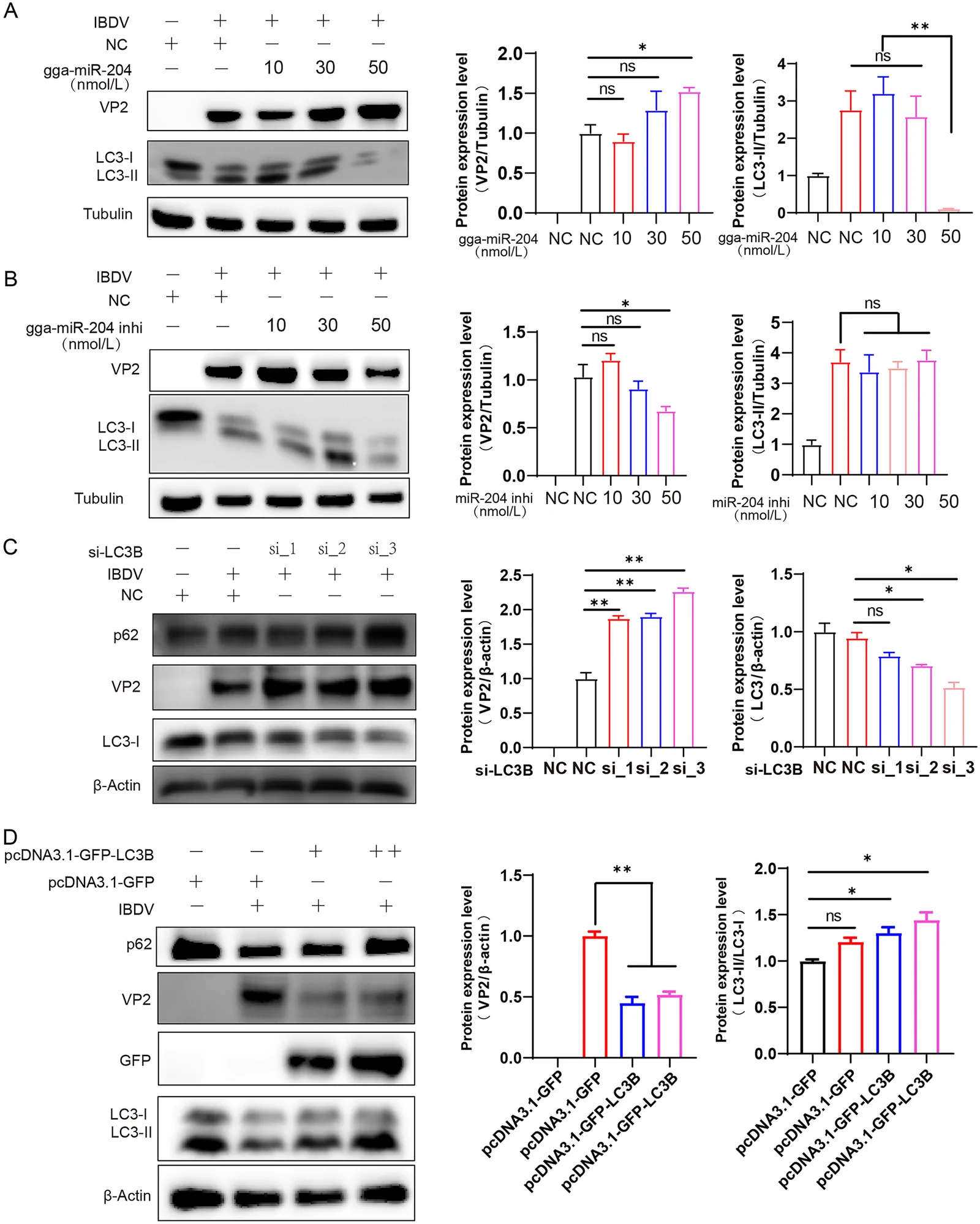
Effects of gga-miR-204 and MAP1LC3B modulation in IBDV-infected HD11 cells. (A, B) HD11 cells were transfected with increasing concentrations of gga-miR-204 mimic (A) or inhibitor (B) and subsequently infected with IBDV. VP2 and LC3 protein levels were analyzed by Western blotting and quantified relative to tubulin. (C) Effects of MAP1LC3B knockdown on p62, LC3, and VP2 protein levels following IBDV infection. (D) Effects of GFP-LC3B overexpression on p62, LC3, and VP2 protein levels following IBDV infection. HD11 cells were transfected with 0.5 μg or 1 μg of GFP-LC3B plasmid DNA per well in 12-well plates before IBDV infection. β-Actin was used as the loading control in C and D. NC, negative control. Data are presented as mean ± SD from three independent experiments. *P < 0.05; **P < 0.01; ns, not significant.

To determine the role of LC3B during IBDV replication, LC3B-specific siRNAs were used to knock down its expression. RT-qPCR analysis showed that si_1, si_2, and si_3 each reduced *MAP1LC3B* mRNA levels by more than 50%, with si_3 showing the strongest knockdown efficiency (Fig. S1). LC3B silencing reduced LC3-II levels and caused p62 accumulation (Fig. 6C). Under these conditions, VP2 protein expression was markedly increased, especially in the si_3 group. Conversely, LC3B overexpression increased LC3-II levels and reduced p62 expression, accompanied by decreased VP2 protein expression (Fig. 6D). Collectively, these results show that gga-miR-204 promotes IBDV replication, whereas LC3B negatively regulates VP2 expression, consistent with a potential contribution of LC3B regulation to the proviral effect of gga-miR-204.

## Discussion

IBDV infection extensively alters host metabolic, stress-response, and autophagy-associated pathways (Song et al., 2025; Wang et al., 2025b). However, systematic approaches for identifying nutrient-responsive miRNAs that functionally regulate cellular susceptibility to IBDV remain limited. In this study, short-term amino acid starvation and glucose starvation were integrated with IBDV infection-associated miRNA profiling to comparatively screen for nutrient-responsive miRNAs potentially involved in viral replication. HD11 cells were selected because this chicken macrophage-like cell line supports IBDV replication and mounts infection-induced innate immune responses (Lee et al., 2015). This model enabled comparative screening and functional validation within the same cellular background. AAS and GS altered LC3-associated molecular phenotypes and reduced IBDV VP2 expression in chicken HD11 macrophages. Small RNA sequencing revealed distinct but partially overlapping miRNA profiles in nutrient-deprived and IBDV-infected cells, with predicted target genes enriched in metabolic, lysosomal, vesicular transport, and stress-response pathways. Among the candidate miRNAs, gga-miR-204 was upregulated by AAS and GS but downregulated following IBDV infection. Functional analyses showed that gga-miR-204 promoted IBDV replication and reduced LC3-II abundance, while reporter assays supported its direct targeting of the chicken *MAP1LC3B* 3′ UTR. *MAP1LC3B* silencing and overexpression increased and decreased VP2 abundance, respectively. These findings support a candidate gga-miR-204–*MAP1LC3B* regulatory relationship in HD11 macrophages, although whether *MAP1LC3B* mediates the proviral effect remains unresolved. This comparative approach provides a starting point for identifying nutrient-responsive miRNAs and investigating their potential roles in linking cellular metabolic state to IBDV replication.Nutrient deprivation affects energy metabolism, protein synthesis, membrane trafficking, and autophagy-related processes (He et al., 2018). Both AAS and GS reduced IBDV VP2 expression while increasing LC3-II abundance and reshaping the host miRNA profile, suggesting that nutrient-responsive miRNAs may participate in LC3-associated stress adaptation. The distinct p62 responses observed under AAS and GS suggest differences in the associated cellular responses, but do not distinguish changes in autophagosome formation from changes in degradation. Accordingly, the changes in LC3-II are more appropriately interpreted as LC3-associated responses rather than definitive evidence of altered autophagic flux. Starvation-responsive miRNAs have previously been shown to regulate autophagy-related genes, as exemplified by miR-495 targeting ATG3 (Li et al., 2016). Notably, gga-miR-204 was induced by nutrient deprivation, whereas its proviral effect was observed in separate functional experiments. This raises the possibility that individual nutrient-responsive miRNAs may exert effects that differ from the overall response to nutrient deprivation. However, whether gga-miR-204 counteracts the reduction in VP2 under nutrient-deprivation conditions was not tested. miR-204 is a conserved miRNA involved in metabolism, autophagy, and viral infection (Huang et al., 2019; Kim et al., 2022). For example, porcine miR-204 inhibits PRRSV replication by targeting LC3B (Yao et al., 2023), whereas chicken miR-204 participates in granulosa-cell autophagy, apoptosis, and lipid metabolism through pathways involving PI3K/AKT/mTOR, TRPM3/AMPK/ULK, and ACSS2 (Chen et al., 2023; Cui et al., 2020). In HD11 cells, gga-miR-204 mimic treatment increased viral RNA abundance and infectious virus production, whereas inhibitor treatment reduced infectious virus production. Together with previous reports, these findings suggest that the effects of miR-204 may vary with the viral and cellular context and support a functional role for gga-miR-204 in IBDV infection in HD11 cells.

The opposite expression responses of gga-miR-204 indicate that nutrient deprivation and IBDV infection regulate this miRNA differently under the conditions examined. Because gga-miR-204 promoted viral replication, its downregulation during infection may help preserve *MAP1LC3B* expression and contribute to host restriction. Alternatively, it may result from infection-stage-dependent changes in miRNA transcription, processing, or stability. Similar to other IBDV-associated miRNAs, such as gga-miR-454, which regulates IFN-β responses through SOCS6 (Fu et al., 2018a; Li and Zheng, 2020), gga-miR-204 may participate in the dynamic balance between host defense and viral permissiveness. Regardless of the mechanism responsible for its infection-associated reduction, gain- and loss-of-function analyses demonstrate that gga-miR-204 abundance substantially influences IBDV replication.

Mechanistically, LC3B is a key ATG8 family member involved in autophagosome formation, membrane remodeling, and immune regulation (Chen et al., 2025b; Jia and Bonifacino, 2020). Dual-luciferase assays demonstrated that gga-miR-204 directly interacted with a specific site in the chicken *MAP1LC3B* 3′ UTR, whereas mutation of this site abolished reporter inhibition. Although gga-miR-204 increased endogenous *MAP1LC3B* mRNA abundance, it reduced LC3-II protein levels, suggesting predominant translational repression accompanied by possible compensatory regulation at the mRNA level. *MAP1LC3B* knockdown reduced LC3-II, increased p62 accumulation, and enhanced VP2 expression, whereas MAP1LC3B overexpression produced the opposite effects. Together, these observations suggest that MAP1LC3B may contribute to the proviral effect of gga-miR-204. However, rescue experiments are required to establish this functional link.

Autophagy-associated processes can exert both proviral and antiviral effects during IBDV infection (Wang et al., 2017; Xu et al., 2021). PD-1-induced autophagy promotes IBDV replication through the PI3K/AKT/FoxO1 pathway (Zhang et al., 2025), whereas IBDV VP3 induces SQSTM1/p62-dependent degradation of TRAF6 and suppresses NF-κB and IFN-β signaling (Deng et al., 2022). IBDV can also exploit an incompletely degradative autophagy–endosomal pathway for viral maturation and release (Wang et al., 2017). Thus, the role of autophagy depends on the cell type, infection stage, lysosomal activity, and specific molecular component involved. The effects of *MAP1LC3B* manipulation on VP2 abundance suggest a potential restrictive role in HD11 cells, highlighting that individual autophagy-related factors may exert effects distinct from the pathway as a whole.

This study combines comparative screening with functional investigation of nutrient-responsive gga-miR-204 and its candidate target *MAP1LC3B*. The findings support the use of nutrient-stress and infection-associated miRNA profiles to identify candidates for functional investigation in HD11 cells, and this approach may be extended to other nutritional conditions. These findings are restricted to the HD11 cell model, and the contribution of gga-miR-204 under nutrient deprivation remains untested; differences in medium composition and the lack of quantitative viability measurements also limit nutrient-specific interpretation. Although reporter assays supported *MAP1LC3B* targeting and *MAP1LC3B* manipulation affected VP2 abundance, its mediation of the proviral effect of gga-miR-204 remains to be established, and the LC3-II and p62 measurements do not demonstrate autophagic flux. Further mechanistic studies and validation in an appropriate IBDV-susceptible B-cell model will help define the broader relevance of these findings.

## Conclusion

This study used comparative profiling of nutrient deprivation and IBDV infection to identify nutrient-responsive miRNAs potentially involved in viral replication. Functional assays showed that gga-miR-204 promotes IBDV replication in HD11 cells. Reporter assays supported its targeting of the chicken *MAP1LC3B* 3′ UTR, while mimic treatment reduced LC3B abundance. *MAP1LC3B* silencing increased VP2 abundance, whereas its overexpression decreased it, although whether *MAP1LC3B* mediates the proviral effect of gga-miR-204 remains unresolved. This approach provides a starting point for investigating nutrient-responsive miRNAs as potential links between cellular metabolic state and IBDV replication.

## Ethics statement

Not applicable.

## Funding

This study was financially supported by the 10.13039/501100012166National Key Research and Development Program of China (2022YFD1800300), the China National Broiler Industry Technology System (CARS-41), the Yangzhou University High-level Talents Support Program.

## Author contributions

Shihao Chen conceived and supervised the study, curated and analyzed the data, and drafted the manuscript. Jiaxing Wang, Qiangzhou Wang, and Zhichao Zhang participated in data analysis and interpretation, performed data validation and visualization, and critically reviewed the manuscript for key intellectual content. Hao Bai developed data analysis software and critically revised the manuscript for important intellectual content. Guobin Chang supervised the study, contributed to the development of data analysis software, and critically revised the manuscript for important intellectual content. All authors have read and explicitly approved the final version of the manuscript for publication and agree to be personally and publicly accountable for all aspects of this work, ensuring that any questions regarding its accuracy or integrity are appropriately investigated and resolved.

## Disclosures

The authors have no conflicts of interest to declare.

## Data Availability

https://ngdc.cncb.ac.cn/gsa/browse/CRA050557

## Footnotes

Supplementary material associated with this article can be found, in the online version, at doi:10.1016/j.psj.2026.108005.

## References

[bib0001] Chen C., Chen W., Ding H., Wu P., Zhang G., Xie K., Zhang T. (2023). High-fat diet-induced gut microbiota alteration promotes lipogenesis by butyric acid/miR-204/ACSS2 axis in chickens. Poult. Sci..

[bib0002] Chen S., Wang J., Wang Q., Ping Y., Yang T., Ma K., Pan S., Bi Y., Bai H., Chang G. (2025). Autophagy-mediated TET2 degradation by ALV-J Env protein suppresses innate immune activation to promote viral replication. J. Virol..

[bib0003] Chen Y., Yi H., Liao S., He J., Zhou Y., Lei Y. (2025). LC3B: a microtubule-associated protein influences disease progression and prognosis. Cytokin. Growth Factor Rev..

[bib0004] Cui Z., Liu L., Kwame Amevor F., Zhu Q., Wang Y., Li D., Shu G., Tian Y., Zhao X. (2020). High expression of miR-204 in chicken atrophic ovaries promotes granulosa cell apoptosis and inhibits autophagy. Front. Cell Dev. Biol..

[bib0005] Deng T., Hu B., Wang X., Ding S., Lin L., Yan Y., Peng X., Zheng X., Liao M., Jin Y., Dong W., Gu J., Zhou J. (2022). TRAF6 autophagic degradation by avibirnavirus VP3 inhibits antiviral innate immunity via blocking NFKB/NF-kappaB activation. Autophagy.

[bib0006] Dulwich K.L., Asfor A., Gray A., Giotis E.S., Skinner M.A., Broadbent A.J. (2020). The stronger downregulation of in vitro and in vivo innate antiviral responses by a very virulent strain of infectious bursal disease virus (IBDV), compared to a classical strain, is mediated, in part, by the VP4 protein. Front. Cell Infect. Microbiol..

[bib0007] Fu M., Wang B., Chen X., He Z., Wang Y., Li X., Cao H., Zheng S.J. (2018). gga-miR-454 suppresses infectious bursal disease virus (IBDV) replication via directly targeting IBDV genomic segment B and cellular suppressors of cytokine signaling 6 (SOCS6). Virus. Res..

[bib0008] Fu M., Wang B., Chen X., He Z., Wang Y., Li X., Cao H., Zheng S.J. (2018). MicroRNA gga-miR-130b suppresses infectious bursal disease virus replication via targeting of the viral genome and cellular suppressors of cytokine signaling 5. J. Virol..

[bib0009] Girdhar K., Powis A., Raisingani A., Chrudinova M., Huang R., Tran T., Sevgi K., Dogus Dogru Y., Altindis E. (2021). Viruses and metabolism: the effects of viral infections and viral insulins on host metabolism. Annu Rev. Virol..

[bib0010] Guo Y., Zhou Z., Jiao H., Gao L., Li X., Cao H., Wang Y., Zheng S.J. (2026). Infectious bursal disease virus suppresses humoral immunity via blocking antibody affinity maturation in chickens. iScience.

[bib0011] He L., Zhang J., Zhao J., Ma N., Kim S.W., Qiao S., Ma X. (2018). Autophagy: the last defense against cellular nutritional stress. Adv. Nutr..

[bib0012] Huang J., Zhao L., Fan Y., Liao L., Ma P.X., Xiao G., Chen D. (2019). The microRNAs miR-204 and miR-211 maintain joint homeostasis and protect against osteoarthritis progression. Nat. Commun..

[bib0013] Jassey A., Wagner M.A., Galitska G., Paudel B., Miller K., Jackson W.T. (2023). Starvation after infection restricts enterovirus D68 replication. Autophagy.

[bib0014] Jia R., Bonifacino J.S. (2020). Regulation of LC3B levels by ubiquitination and proteasomal degradation. Autophagy.

[bib0015] Kim S., Lee I., Piao S., Nagar H., Choi S.J., Kim Y.R., Irani K., Jeon B.H., Kim C.S. (2022). miR204 potentially promotes non-alcoholic fatty liver disease by inhibition of cpt1a in mouse hepatocytes. Commun. Biol..

[bib0016] Lee C.C., Wu C.C., Lin T.L. (2015). Role of chicken melanoma differentiation-associated gene 5 in induction and activation of innate and adaptive immune responses to infectious bursal disease virus in cultured macrophages. Arch. Virol..

[bib0017] Li C., Sun L., Lin H., Qin Z., Tu J., Li J., Chen K., Babu V.S., Lin L. (2019). Glutamine starvation inhibits snakehead vesiculovirus replication via inducing autophagy associated with the disturbance of endogenous glutathione pool. Fish. Shellfish. Immunol..

[bib0018] Li J., Zheng S.J. (2020). Role of MicroRNAs in host defense against infectious bursal disease virus (IBDV) infection: a hidden front line. Viruses.

[bib0019] Li W., Yang Y., Hou X., Zhuang H., Wu Z., Li Z., Guo R., Chen H., Lin C., Zhong W., Chen Y., Wu K., Zhang L., Feng D. (2016). MicroRNA-495 regulates starvation-induced autophagy by targeting ATG3. FEBS Lett..

[bib0020] Ma X., Tian Y., Zhang W., Zhang R., Xu X., Han J., Jiang Y., Wang X., Man C. (2023). Stress-induced immunosuppression inhibits immune response to infectious bursal disease virus vaccine partially by miR-27b-3p/SOCS3 regulatory gene network in chicken. Poult. Sci..

[bib0021] Mukherjee K., Ghoshal B., Ghosh S., Chakrabarty Y., Shwetha S., Das S., Bhattacharyya S.N. (2016). Reversible HuR-microRNA binding controls extracellular export of miR-122 and augments stress response. EMBO Rep..

[bib0022] Niu X., Han J., Huang M., Wang G., Zhang Y., Zhang W., Yu H., Xu M., Li K., Gao L., Wang S., Chen Y., Cui H., Zhang Y., Liu C., Wang X., Gao Y., Qi X. (2024). Infectious bursal disease virus VP5 triggers host shutoff in a transcription-dependent manner. mBio.

[bib0023] Onishi K., Shibutani S., Goto N., Maeda Y., Iwata H. (2019). Amino acid starvation accelerates replication of Ibaraki virus. Virus. Res..

[bib0024] Song C., Li S., Yuan Y., Gao W., Qiu X., Tan L., Sun Y., Tang N., Qu Y., Liao Y., Ding C. (2025). Multi-omics profiling reveals infectious bursal disease virus-induced alterations in gene expression and metabolism in chicken bursa of fabricius. Vet. Microbiol..

[bib0025] Wang J., Wang Q., Ping Y., Huang X., Yang T., Bi Y., Chang G., Chen S. (2025). Identification and characterization of chicken TRIM45 and its role as a negative regulator of ALV-J replication in vitro. Avian Pathol..

[bib0026] Wang J., Wang Q., Ping Y., Huang X., Yang T., Liu Z., Bai H., Chang G., Chen S. (2025). Transcriptomic analysis identifies key genes associated with nutrient deprivation responses in infectious bursal disease virus infection. BMC. Genomics..

[bib0027] Wang Q., Wu W., Cai Q., Wang J., Gao Q., Iftikhar M., Bi Y., Bai H., Gu L., Chang G., Chen S. (2025). The role of miRNAs in chicken immune regulation and prospects for disease-resistant breeding. Anim. Res. One Health..

[bib0028] Wang Y., Duan Y., Han C., Yao S., Qi X., Gao Y., Maier H.J., Britton P., Chen L., Zhang L., Gao L., Gao H., Shen N., Wang J., Wang X. (2017). Infectious bursal disease virus subverts autophagic vacuoles to promote viral maturation and release. J. Virol..

[bib0029] Wu Y., Wen J., Han J., Tian Y., Man C. (2022). Stress-induced immunosuppression increases levels of certain circulating miRNAs and affects the immune response to an infectious bursal disease virus vaccine in chickens. Res. Vet. Sci..

[bib0030] Xu C., Li T., Lei J., Zhang Y., Zhou J., Hu B. (2021). The autophagy cargo receptor SQSTM1 inhibits infectious bursal disease virus infection through selective autophagic degradation of double-stranded viral RNA. Viruses.

[bib0031] Yao Y., Li S., Zhu Y., Xu Y., Hao S., Guo S., Feng W.H. (2023). miR-204 suppresses porcine reproductive and respiratory syndrome virus (PRRSV) replication via inhibiting LC3B-mediated autophagy. Virol. Sin..

[bib0032] Zhang Q., Yue F., Sun G., Jiang L., Li P., Zhu Y., Liu Z., Zhu Y., Niu R., He H., Sun Z., Wang X. (2025). PD-1 induces autophagy via the PI3K/AKT/FoxO1 pathway to promote infectious bursal disease virus replication. Front. Immunol..

[bib0033] Zhang T., Wang S., Liu Y., Qi X., Gao Y. (2023). Advances on adaptive immune responses affected by infectious bursal disease virus in chicken. Front. Immunol..

[bib0034] Zhang W., Wang X., Gao Y., Qi X. (2022). The over-40-years-epidemic of infectious bursal disease virus in China. Viruses.

