# Supplementary material for: Comparative screening identifies gga-miR-204 as a nutrient-responsive miRNA that targets MAP1LC3B and promotes infectious bursal disease virus replication in HD11 cells
Source: Poult Sci. 2026 Oct 21;105(12):108005. doi: 10.1016/j.psj.2026.108005 (PMC13735245; doi:10.1016/j.psj.2026.108005)
Supplement: Supplementary file 1 [file mmc1.docx]

**Figure S1. Validation of LC3B knockdown efficiency.** Relative LC3B mRNA expression was assessed in HD11 cells transfected with a negative control siRNA (si-LC3B NC) or three independent LC3B-targeting siRNAs (si 1, si 2, and si 3). LC3B mRNA levels were normalized to actin mRNA levels. ns, not significant; **P* < 0.05; ***P* < 0.01.
